# Merkel cell carcinoma of the upper extremity: Case report and an update

**DOI:** 10.1186/1477-7819-6-32

**Published:** 2008-03-07

**Authors:** Michail Papamichail, Ioannis Nikolaidis, Nicolas Nikolaidis, Chryssoula Glava, Ioannis Lentzas, Konstantinos Marmagkiolis, Kriton Karassavsa, Michail Digalakis

**Affiliations:** 1 General Hospital of Athens, ''Asklipion Voulas", Athens, Greece; 2 Tzaneion General Hospital, Piraeus, Greece; 3 Aberdeen Royal Infirmary Hospital, Aberdeen, UK; 4 Montreal Heart Institute, Montreal QC, Canada

## Abstract

**Background:**

Merkel cell carcinoma is a rare but aggressive cutaneous primary small cell carcinoma. It is commonly seen in elderly affecting the head, neck, and extremities. Macroscopically may be difficult to distinguish MCC from other small cells neoplasms especially oat cell carcinoma of the lung.

**Case presentation:**

It is presented a case report concerning a 72 years old male with a MMC on the dorsal aspect of the right wrist. The patient underwent a diagnostic excisional biopsy and after the histological confirmation of the diagnosis a second excision was performed to achieve free margins. No postoperative radiation or adjuvant chemotherapy was given and within 9 years follow up no recurrence was reported.

**Conclusion:**

Although most cases present as localized disease treatment should be definitive due to high rates of local or systemic recurrence. Treatment includes excision of the lesion, lymphadenectomy, postoperative radiotherapy and chemotherapy depending on the stage of the disease. Even when locoregional control is achieved close surveillance is required due to high rates of relapse.

## Background

Merkel cell carcinoma (MCC) is a rare cutaneous malignancy that was first described by Toker in 1972 [1]. This rare aggressive neoplasm is thought to originate from the neurocrest derivatives round shaped Merkel cells located in the basal layer of the epidermis and containing neurosecretory granules [2-5].

Although aetiology is not fully illuminated, there are several risk factors that contribute to its pathogenesis. Those include UV light, sun-related skin malignancies (Squamous Cell Carcinoma, Basal Cell Carcinoma), psoriasis treatment with methoxsalen and arsenic exposure. Patients on immunosuppressive agents or patients with diagnosis of AIDS, chronic lymphocytic leukemia, congenital dysplasia syndrome and organ recipients carry a higher risk as well [6-11].

Clinically, MCC appears as a painless, firm, non tender, ulcerated skin lesion commonly less than 2 cm in size at the time of presentation [4,8]. Most cases present as localized disease (70%–80%) followed by regional lymph node involvement (9%–26%) and distant metastasis (1%–4%) [8]. These characteristics often raise the suspicion of a skin malignancy but confirmation of diagnosis is made by excisional biopsy. The differential diagnosis of MCC from other small cells neoplasms can be difficult, even on histological examination [10]. For definitive diagnosis in these cases, electron microscopy is necessary [5].

## Case presentation

**Figure 1:**
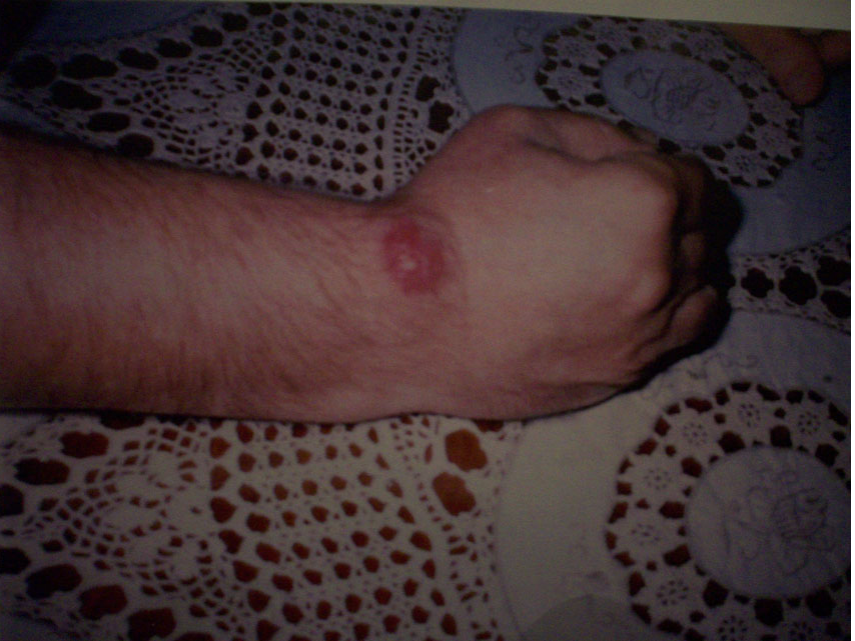
Macroscopic view of the lesion

A 72-year-old male presented in December 1998 with a painless nodular, red and firm 2 cm plaque located on the dorsal aspect of the right wrist (Figure 1) noticed 1–2 months before. No history of previous skin lesions elsewhere was reported.

**Figure 2:**
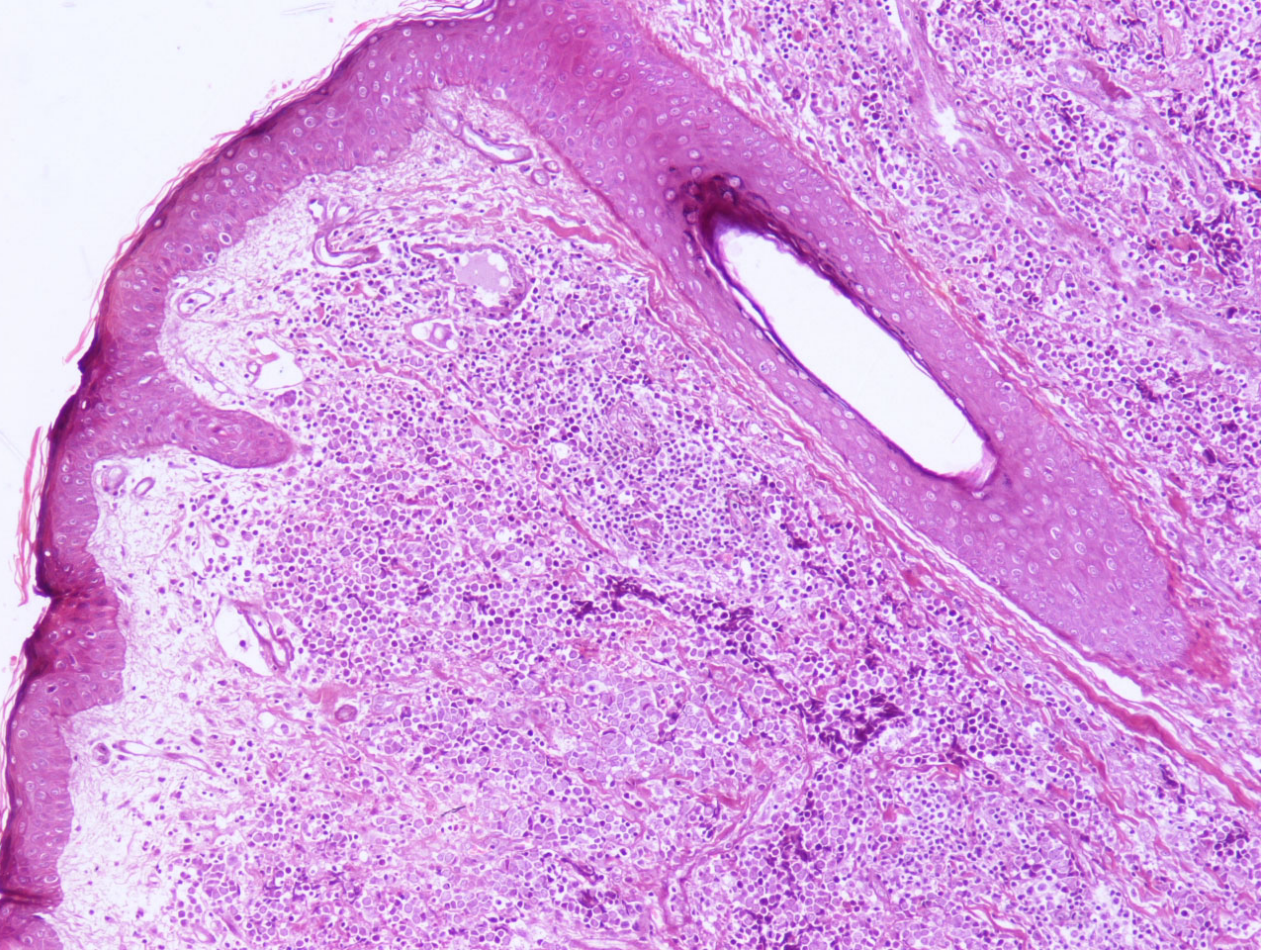
H-E x 100

**Figure 3:**
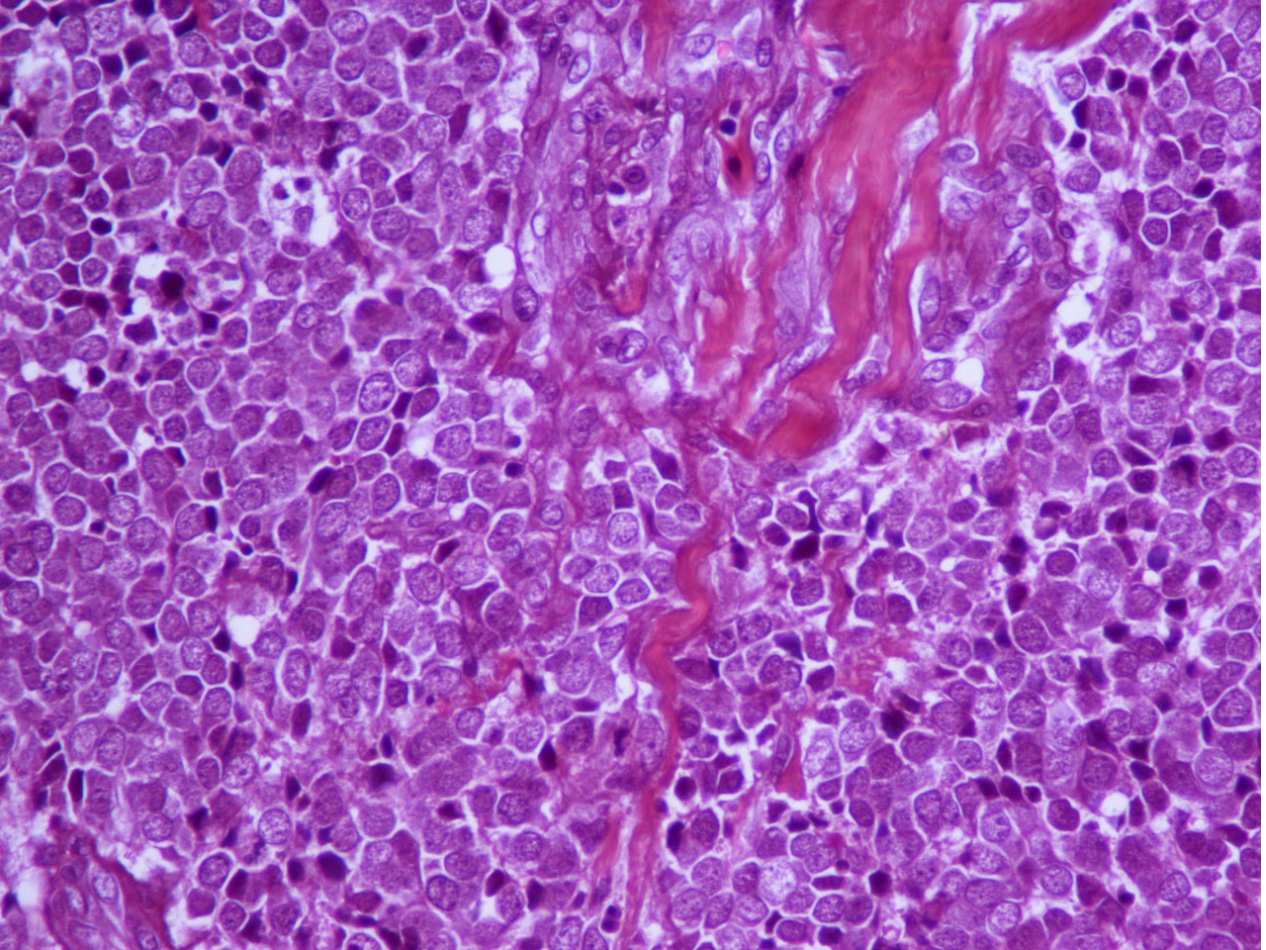
H-E x 400

**Figure 4:**
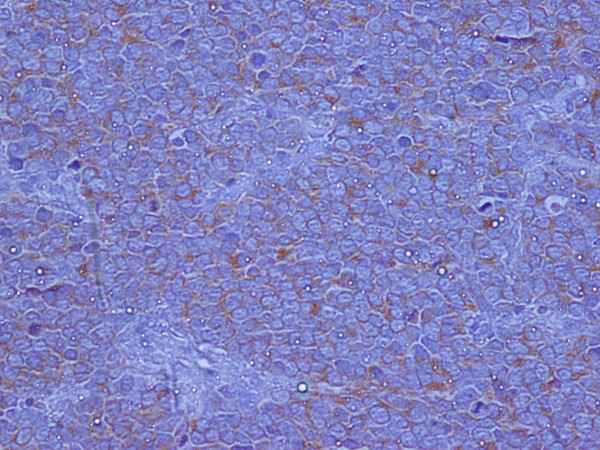
EMA x 400

**Figure 5:**
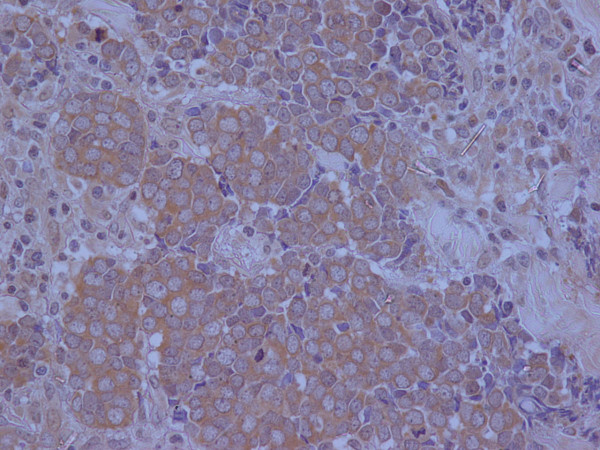
NSE x 400

An excisional biopsy was performed. Microscopical examination of the lesion revealed the invasion of dermis and subcutaneous tissue by a small cell solid tumor with diffuse pattern of infiltration (Figure 2). The excisional margins were positive although dermal lymphatics were intact and no exceeding to the adjacent structures such us, veins, tendons or nerves was discovered. The tumor cells were small, with scanty acidophilic cytoplasm, round vescicular nuclei and multiple nucleoli (Figure 3). Mitotic figures were numerous. In immunohistochemical examination, the tumor cells showed diffuse positivity for Epithelial Membrane Antigen (EMA, Figure 4) and Neuron Specific Antigen (NSE, Figure 5). Lymphatic Common Antigen (LCA), Thyroid Transcription Factor – 1 (TTF-1) and CD99 were negative. Based on to these histological and immunohistochemical features, diagnosis of Merkel Cell Tumor was established.

**Figure 6:**
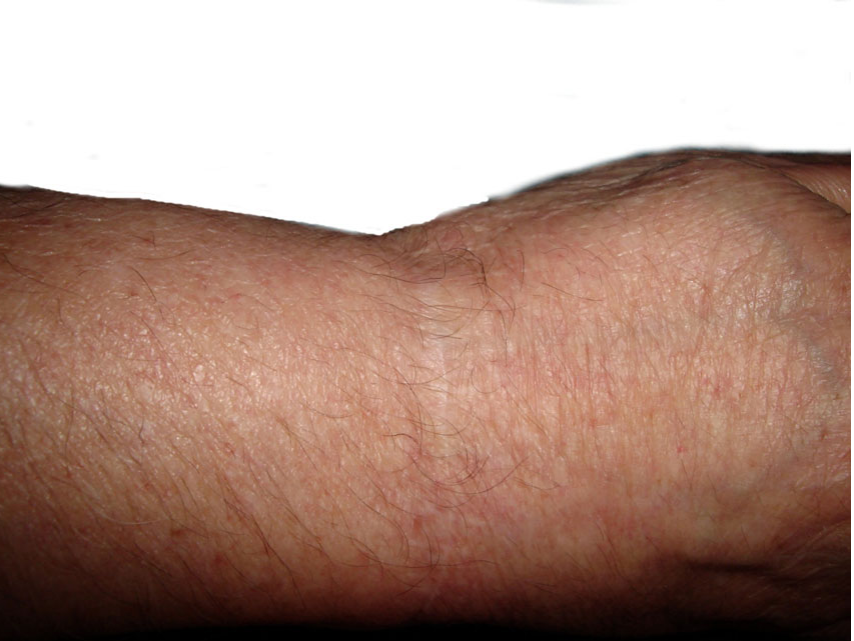
9 years post-op

The patient underwent an imaging evaluation with a CT scan for staging. The CT did not reveal any masses, lymphadenopathy or distant metastases. An additional excision was performed in order to achieve approximately margins 2–3 cm wide and 1–2 cm deep. The patient expressed the willing not to receive postoperative radiation or adjuvant chemotherapy which was justified based on the stage of the disease and the cardiovascular and pulmonary co-morbidities. We scheduled CT imaging follow up every 6 months for the first 3 years and then annually for the upcoming years. No recurrence was reported until April 2007. (Figure 6).

## Discussion

MMC is an aggressive neoplasm with an overall unfavourable prognosis [12], therefore it requires definite treatment. It usually occurs in older patients with less than 5% cases seen before the age of 50 years and it has an annual incidence of 0.42 per 100.000. Both sexes are affected with a male predominance, although in some series higher incidence in women is reported [4,8,9]. Higher likelihood is reported in whites and it affects sun exposed areas such as head and neck (50%), upper and lower limbs (35%–40%) and less than 10% in the trunk [8]. It has also been reported that MMC rarely can occur on anatomic sites such as vulva, penis, pharynx and oral or nasal mucosa. [7].

Macroscopically, MCC appears as a nodular, sometimes ulcerated skin lesion with a reddish or violaceous hue [12]. Microscopically, the tumor is centered in the dermis or sometimes in the subcutaneous tissue, with the overlying epidermis being usually not involved [13]. The tumor cells are small and round, disposed in a diffuse or, rarely, trabecular architectural pattern [14,15]. The cytoplasm is scanty, visible as a thin eosinophilic rim. The nuclei are round and vescicular, with a typically fine granular chromatin, multiple nucleoli and numerous mitotic figures. The tumour stroma contains abundant vessels with hypertrophic endothelial cells. [15,16]

Immunohistochemically, the tumor cells are usually positive for low-molecular-weight cytokeratin (CK AE1), predominantly cytokeratin 20 (CK20) [17], neurofilaments and NSE [18]. Additionally to these markers, some cases of MCC have shown focal reactivity for chromogranin, synaptophysin, vasoactive intestinal peptid, pancreatic polypeptide, calcitonin, substance P, somatostatin, ACTH, other peptide hormones and CD117. [19-24]

Differential diagnosis has to be made between MCC and other small cell neoplasms (small cell neuroendocrine lung carcinoma, malignant lymphoma, Ewing's sarcoma/PNET category). Sometimes, tumors with an appearance identical to pulmonary small cell neuroendocrine carcinoma are found in the skin. [12] The consistent positivity of the MCC for CD20 and the negativity for TTF-1 are important in the differential diagnosis from small cell neuroendocrine lung carcinoma [25-27]. The monotonous nature of the dermal round cell infiltrate and the diffuse pattern of infiltration are responsible for MCC's misdiagnosis as malignant lymphoma [28]. Differential diagnosis in this case is made using the immunohistochemical lymphatic marker LCA. Finally, differential diagnosis of MCC from PNET is base on the negativity of the neoplastic Merkel cells for CD99, positive in Ewing's sarcoma/PNET [29].

The fact that MCC can be seen in association with in situ or invasive SCC, with duct-like structures of eccrine type, and with basal call carcinoma-like areas suggests that it originates from a potential stem cell of ectodermal derivation. [30-33]

Chromosomal abnormalities localized on the short arm of chromosome 1, associated with Merkel cell tumor are common in melanoma and neuroblastoma. Chromosomal abnormalities (loss of heterozygosis in chromosome 3p21) associated with small cell lung neuroendocrine carcinoma is related to Merkel cell carcinoma as well. [8].

Due to its rarity and the lack of cases for a randomized prospective trial no consensus of the appropriate treatment protocol for MCC is made so far [6-8]. Therapeutic options depend on the stage of the disease at the time of presentation whereas the most important prognostic factor is the absence of nodal involvement [34].

Surgery remains the gold standard for localized disease and is considered to be successful when margins 3 cm wide and 2 cm deep are achieved [8,34]. Some controversy exists showing that when the tumour size is less than 1.5–2 cm, obtaining margins less than 2–3 cm did not lead to higher recurrences rates. [11] Mohs micrographic surgery is an alternative method of wide clearance, especially on sites required excellent cosmetic results [6] and some studies report better rates of locoregional control [8,10,35,36]. A benefit of this method is the better inspection of all major borders of the lesion. [7,36]

Postoperative radiotherapy in node free patients either discovered clinically, with imaging techniques, with a negative sentinel node biopsy, or after routine nodal dissection still remains controversial. Due to the high rates of local relapse, routine use of 45–60 Gy [8,10] to the area of the lesion has been found to decrease local recurrence [36]. Other series showed no significant difference compared with surgery only [11] and distant metastasis and overall survival seem to be similar compared to those who did not receive radiation [10,37]. Postoperative radiotherapy could be beneficial in cases of large primary tumours or unattainable free surgical margins due to cosmetic or functional difficulties [4,8] but radiating permanent margins did not yield satisfactory results [34].

Many authors advocate that lymph node recurrence often represents the delayed manifestation of pre-existing occult micrometastases rather than inadequate local control of primary tumour [11]. Based on this, sentinel node biopsy should be strongly considered. [11]. Involvement of the regional lymph nodes decreases dramatically the survival rates (88% to 50%) and it appears in 50%–70% of all patients within 2 years by the time of diagnosis [38]. Other poor prognostic factors are tumour size >2 cm, male sex, age >60 years, immunosuppression and location on lower extremities [7-9,36]. Due to this high rate of spreading, prophylactic nodal clearance of free disease nodes is advocated in order to improve outcome. In some studies sentinel node status was evaluated and a sentinel node biopsy was performed in order to identify occult micrometastases, showing low relapsing rates [6,11,38]. However, sentinel node biopsy is not attempted if additional therapy is not tolerated by the patient [11]. Based on an another study it has been recommended prophylactic lymphadenectomy only in patients with lesions present for longer than 6 weeks prior seeking medical advise or when tumour exceeds 1.5 cm in size. [10] Many authors advocate the routine lymph node dissection, including or not sentinel node biopsy [7,34] but others conclude that routine lymph node dissection improves locoregional control but has no effect on survival [39].

When nodal infiltration is established, definite management includes complete lymphadenectomy and postoperative radiotherapy. As a result of increased rate of recurrence, even when lymph nodes have been removed, strict follow-up is required. [8,10,38].

Disseminated disease whether primary or recurrent has a very poor prognosis with an average expected survival of 8 months by the time of diagnosis. Imaging techniques such as CT, MRI, PET scan and ocrteotide schintigraphy have all been used to detect regional or distant metastases. [7,11] Regional metastases are common, and distant metastases can also occur, particularly in liver, bone, lung, brain and skin. Rare cases of distant metastases of MCC in bone marrow, pleura, testis, small bowel and stomach have been reported [5,8,37]. Treatment in case of MCC with distal metastases consists of palliative radiotherapy and chemotherapy. Multiple agents have been used with different response rates [38]. Those include cyclophosphamide, doxorubicin, etoposide, cis-platinum, vincristine, methotrexate, 5-fluorouracile, carboplatinum [8,34]. Biologic agents such as interferon, tumour necrosis factor (TNF) and imatinib mesylate promise better results on local (TNF) or systemic control of MCC. [7] Radiotherapy can be used as palliative therapy of cutaneous deposits or bone and brain metastases [8]. Patients developing recurrence within the radiotherapy field are not candidates for further high dose radiotherapy (>50 Gy) [9].

## Conclusion

The overall 5-year survival rate for patients with Merkel cell carcinoma is 50% to 68% [38]. Considering the high incidence of local recurrence (27%–60%) regional node involvement (45%–91%) or distant metastases (18%–52%) [8], treatment should be definite with close follow up. Despite the aggressiveness of MCC, early diagnosis, optimal resection with clear margins and postoperative radiotherapy achieve loco regional control of the tumor and long term survival, although radiotherapy still remains controversial [40]. In the cases of lymph node involvement, prognosis is less favourable considering that despite nodal dissection and adjuvant radiotherapy the majority of patients will ultimately develop distant metastases.

## Competing interests

The author(s) declare that they have no competing interests.

## Authors' contributions

**MP**: drafted the article; **LN**: helped in drafting the article; **NN **helped in drafting the draft **CG **carried out the immunoassays; **LL**: participated in the design of the study and performed the statistical analysis; **MK**: conceived of the study, and participated in its design and coordination and helped to draft the manuscript. **KK**: conceived of the study, and participated in its design and coordination and helped to draft the manuscript. **MD**: Supervised the preparation of the article and helped in preparation of final manuscript.

All authors read and approved the final manuscript.
